# Dextran-Cholesterol Carrier Encapsulated Efficient Photosensitizer for the Photodynamic Killing of Cancer Cells

**DOI:** 10.3390/molecules28114404

**Published:** 2023-05-28

**Authors:** Biru Wu, Zhuoheng Gan, Shengchang Tao, Qiang Wang, Yuchen Song, Hua Zhong, Fang Hu

**Affiliations:** 1 Biomaterials Research Center, School of Biomedical Engineering, Southern Medical University, Guangzhou 510515, China; 2 Department of Pharmacy, Affiliated Dongguan Hospital, Southern Medical University, Dongguan 523059, China; 3 Department of Orthopaedics, The Fifth Affiliated Hospital, Southern Medical University, Guangzhou 510900, China; 4 Division of Laboratory Medicine, Zhujiang Hospital, Southern Medical University, Guangzhou 510282, China

**Keywords:** PDT, dextran, heavy atom

## Abstract

Selective photodynamic therapy (PDT) for cancer cells is more efficient and much safer. Most selective PDTs are realized by antigene-biomarker or peptide-biomarker interactions. Here, we modified dextran with hydrophobic cholesterol as a photosensitizer carrier to selectively target cancer cells, including colon cancer cells, and fulfilled selective PDT. The photosensitizer was designed with regular Aggregation-Induced Emission (AIE) units, including triphenylamine and 2-(3-cyano-4,5,5-trimethylfuran-2-ylidene)propanedinitrile. The AIE units can help to decrease the quenching effect in the aggregate state. The efficiency of the photosensitizer is further improved via the heavy atom effect after bromination modification. We found that the obtained photosensitizer nanoparticles could selectively target and ablate cancer cells after encapsulation into the dextran-cholesterol carrier. This study indicates that the polysaccharide-based carrier may have potential for cancer-targeting therapy beyond expectations.

## 1. Introduction

Colon cancer, a prevalent malignant disease in the gastrointestinal tract with rising incidence worldwide, is primarily treated with surgery and chemotherapy currently. The instability of small molecule drugs in the acidic gastrointestinal tract and their low tumor selectivity make it difficult to achieve the desired local chemotherapy effect. To address this issue, most treatment systems encapsulate drugs within nanocarriers [1,2]. Nanoparticles (NPs) help minimize premature drug leakage and enable drugs to target and accumulate at tumor sites due to the enhanced penetration and retention (EPR) effects that arise from the vascular leakage of the tumor system [3]. Additionally, NPs are stable colloidal polymers that can prevent the rapid digestion of drugs in the gastrointestinal tract during transit.

Polysaccharide-based nanocarriers are natural polymers with good biocompatibility and biodegradability that have been extensively devoted to manufacturing NPs in recent years. Since the polysaccharide matrices are mainly fermented by the exclusive intestinal micro-organisms rather than degraded into inactive ingredients by enzymes, the polysaccharide-based NPs can provide a longer circulating half-life, and this guarantees that the drug release is transmitted sufficiently to the colon. As a result, polysaccharides are regarded as a promising nanocarrier for carrying anticancer drugs for the therapy of colon cancer [4,5]. For example, previous studies successfully attempted to utilize pullulan with hydrophobic modification of acetic anhydride to prepare paclitaxel-incorporated NPs for colon cancer treatment [6]. Additionally, a paclitaxel-coated injectable hydrogel based on Pinus koraiensis polysaccharide was developed to restrain tumor growth with diminished body toxicity [7]. Chiu et al. successfully developed solid lipid NPs, whose surfaces were sequentially coated with folic acid and dextran layer by layer while the interior was loaded with DOX and superparamagnetic iron oxide NPs, in order to achieve dual targeting formulations and accumulation in colon tumors for chemo/magnetothermal combination therapy [8]. Mukhopadhyay et al. labeled chitosan-biotin on NPs to achieve accumulation in cancer cells through receptor-mediated endocytosis and the release of pH-triggered core agents, which successfully realized selectivity between normal cells and cancer cells and effectively killed various cancer cells [9].

Dextran is a homopolysaccharide composed of glucose with low toxicity, chemical stability, thermal stability, and extended-release properties [5]. Moreover, dextranase is a dextran hydrolase produced by bacteria specifically surviving in the colon. Dextranase has an association with dextran. The excess dextranase in the colon can result in the exclusive binding of the dextran with colon cells. Thus, the dextran derivates are good candidates to design colon cell-targeting carriers [8]. It has formerly been reported that hSET1 antisense and SN38 anticancer were encapsulated in NPs based on cysteine trimethyl chitosan and carboxymethyl dextran to cure colorectal cancerous cells [10]. Therefore, dextran-based carriers are promising to deliver medications to the colon cells.

Photodynamic therapy (PDT) has attracted substantial attention because of its noninvasive treatment, minimal side effects, and spatiotemporal selectivity via regulating exposure, which enables it to become a potential therapeutic method [11]. The mechanism of PDT involves photochemical reactions between photosensitizers (PSs) excited by light and cellular substrates or molecular oxygen to produce energy, bringing about reactive oxygen species (ROS)-mediated cell death [12]. PSs are the striking factor of PDT. ROS generation requires the interaction of cellular substrates or molecular oxygen with the PSs of triplet excited state to effectively generate reactive oxygen species (ROS). Distinguished PSs can more effectively carry out the intersystem crossing (ISC) process to efficiently generate reactive oxygen species (ROS). Considerable studies have shown that the heavy atom effect can increase the degree of spin-orbit coupling of molecules to enhance ISC and increase ROS generation efficiency [13,14,15]. Most heavy atom-modified PSs are hydrophobic. Hydrophobic PSs tend to aggregate in an aqueous solution and cannot be used directly [16,17]. They are usually used in NPs format with good water dispersity [18,19,20]. On account of this, the PSs encapsulated in the dextran-based carriers are promising for targeted PDT treatment of colon disease.

In this paper, dextran was modified with hydrophobic cholesteryl hemisuccinate (CHS) to generate amphiphilic dextran-CHS as the colon cancer cell-targeting carrier, a donor-acceptor structure-based PS was then modified with bromine atoms to increase the efficiency, and the PS was encapsulated into dextran-CHS to form NPs to realize selective PDT of colon cells. The PSs named TCF with triphenylamine as donor and 2-(3-cyano-4,5,5-trimethylfuran-2-ylidene)propanedinitrile as acceptor, and its brominated derivative TCFBr obtained by incorporating two bromine atom on the acceptor unit of TCF was synthesized and characterized. Furthermore, the influences of bromination on the physicochemical properties of these two PSs and their NPs, such as singlet oxygen (^1^O_2_) and total ROS, were assessed, and the therapeutic efficacy of TCFBr NPs on colon cancer cells and normal cells was also evaluated in vitro.

## 2. Results and Discussion

### 2.1. Synthesis and Characterization of Dextran-CHS

**Figure 1:**
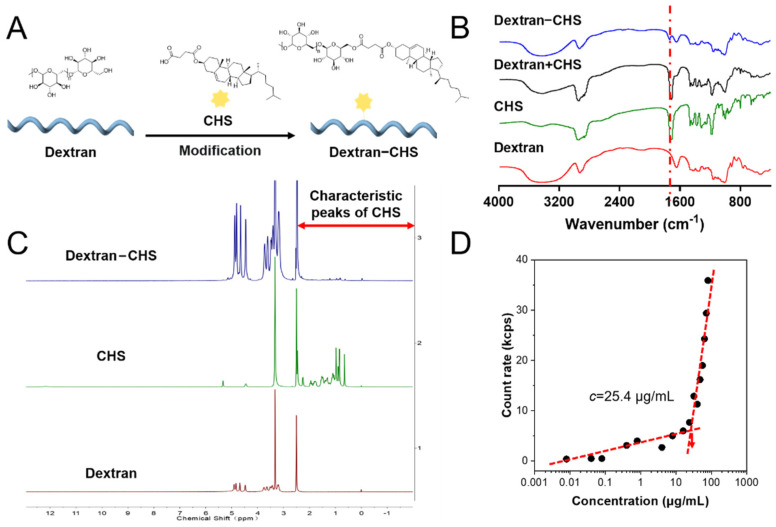
Synthesis and characterization of dextran-CHS. (**A**) Preparation of dextran-CHS. (**B**) Infrared spectra of dextran, CHS, dextran + CHS, and dextran-CHS made by the potassium bromide tableting. (**C**) ^1^H NMR spectrum of dextran, CHS, and dextran-CHS in *d*-chloroform. (**D**) Critical micelle concentration (CMC) of TCFBr NPs estimated by mutational particle-derived count rate.

Dextran, a hydrophilic non-ionic molecule with good biocompatibility that can be easily converted into amphiphilic polymers that can self-assemble in an aqueous solution to form nano micelles or NPs, can be employed as a drug carrier after hydrophobic modification [21]. CHS is a lipid-soluble and biocompatible cholesterol derivative that is widely used in integral membrane protein crystallization [22,23,24]. 1-(3-dimethylaminopropyl)-3-ethylcarbodiimide hydrochloride (EDC.HCl) and 4-dimethylaminopyridine (DMAP) were used to catalyze the reaction of CHS and dextran, allowing CHS to be grafted onto a glucose molecular chain to obtain amphiphilic dextran-CHS (Figure 1A) [25]. The infrared spectra of samples, including dextran, CHS, dextran-CHS, and dextran plus CHS, were measured to verify the ligation of dextran to CHS. It can be observed that dextran-CHS owned an obvious ester carbonyl group (C=O) stretching vibration peak at 1730 cm^−1^, indicating the successful linkage of CHS to the glucose backbone (Figure 1B) [26]. As shown in Figure 1C, we also measured the ^1^H NMR spectra of these samples using DMSO-*d*_6_ as the solvent to further verify the linkage. ^1^H NMR spectra of dextran-CHS not only had the peaks with δ 4.40–4.80 appear at ^1^H NMR spectra of dextran but also possessed the characteristic peaks of methylene (CH_2_) with *δ* 1.21 and methyl (CH_3_) with *δ* 0.85 exist in ^1^H NMR spectra of CHS, suggesting that the CHS were successfully grafted. According to the chemical shift of the proton peak assigned in Figure S16, it can be determined that dextran-CHS contained both dextran and CHS peak. After CHS grafting, the critical micelle concentration (CMC) value of dextran-CHS was 25.4 μg/L by calculating the inflection point of the straight line simulated by the particle-derived count rate of dextran-CHS with different concentrations affected by micelle formation. This means that the amphiphilic dextran-CHS can self-assemble to aggregate in water at a low concentration.

### 2.2. Enhanced Photosensitization by Bromination

**Figure 2:**
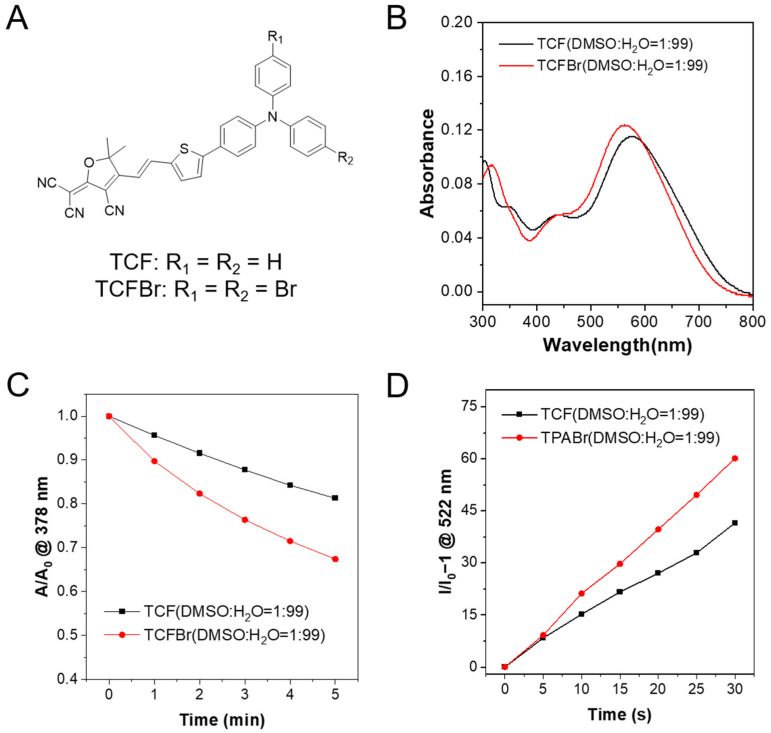
Absorption properties and ROS-producing capacity. (**A**) Chemical structures of TCF and TCFBr. (**B**) Absorption spectra of TCF and TCFBr in the DMSO/water (1/99, *v*/*v*) solution. ^1^O_2_-producing capacity (**C**) and ROS-generating capacity (**D**) of TCF and TCFBr under green light (530 nm, 60 mW/cm^2^) excitation were evaluated by ABDA (50 μmol/L) and DCFH (0.15 μg/mL), respectively. A_0_ and A are the absorbances of ABDA at 378 nm. I/I_0_ − 1 represents the relative fluorescence intensity of DCF at 522 nm with different irradiation time. [TCF and TCFBr] = 5 μmol/L.

The synthesis routes of the designed PSs are shown in Scheme S1. The compounds were effortlessly synthesized by the Suzuki coupling reaction and Knoevebagel condensation reaction between 2-(3-cyano-4,5,5-trimethylfuran-2-ylidene)propanedinitrile and aldehydes [27]. ^1^H NMR and ^13^C NMR displaced in Figures S8–S15 were then measured to identify the structures of synthesized TCF, TCFBr, and intermediate products. The optical properties of TCF and TCFBr are depicted in Figure 2. With push–pull electronic structures, the maximum absorption peaks of TCF and TCFBr aggregates in water are 576 nm and 562 nm, respectively. The addition of two bromine atoms to the donor makes the absorption peak of TCFBr blue-shifted by 14 nm compared to TCF. The expectation of adding bromine atoms is to enhance ISC through the heavy-atom effect, which can lead to the increased spin-orbital coupling to promote ISC [28,29,30,31].

9,10-Anthracenediyl-bis(methylene)dimalonic acid (ABDA) is a ^1^O_2_ indicator that can be degraded by cycloaddition reaction with ^1^O_2_ to engender the corresponding endoperoxide product [32], so the ability of PSs to generate ^1^O_2_ is relevant to its efficiency in degrading ABDA. In the presence of TCF or TCFBr, ABDA undergoes obvious photobleaching upon light irradiation (530 nm, 60 mW/cm^2^) because of their reaction with ^1^O_2_, which is generated by TCF or TCFBr upon laser irradiation (Beijing Laserwave OptoElectronics Technology Co., Ltd., Beijing, China) (Figure S1). The absorption is linearly related to the concentration, so it is reliable to compare the ^1^O_2_ production rate of PSs by monitoring the absorption decrease of ABDA at 378 nm after the different exposure time (Figure 2C). At the same molar concentration, TCF decomposed 18.7% of ABDA while TCFBr decomposed 32.6% within 5 min of laser irradiation. The efficiency of TCFBr to degrade ABDA increases to 1.74 times that of TCF, illustrating that PSs tuned by bromine atoms are more productive due to the heavy atom effect.

2′,7′-dichlorodihydrofluorescein (DCFH) is widely used as a fluorescent probe to detect the total ROS-producing capacity, since it can be oxidized by ROS into 2′,7′-dichlorofluorescein (DCF). The ratio of fluorescence intensity changing at 522 nm under different irradiation times can be used to determine the efficiency of ROS generation by PSs, which also showed that the total ROS generation ability, TCFBr, was superior to TCF (Figure 2D and Figure S2).

**Figure 3:**
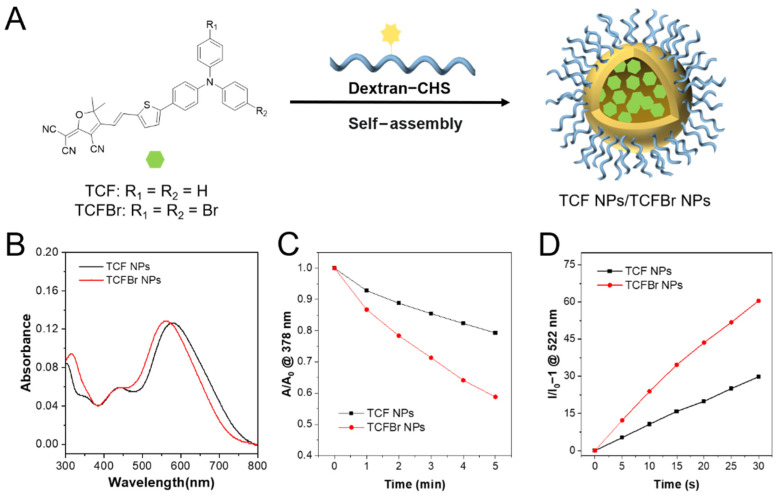
Optical properties of NPs. (**A**) Schematic synthesis of TCF NPs and TCFBr NPs. (**B**) Absorption spectra of TCF NPs and TCFBr NPs in water solution. ^1^O_2_ production capacities (**C**) and ROS-generating capacity (**D**) of TCF NPs and TCFBr NPs under green light (530 nm, 60 mW/cm^2^) excitation were evaluated by ABDA (50 μmol/L) and DCFH (0.15 μg/mL), respectively. A_0_ and A are the absorbances of ABDA at 378 nm. I/I_0_ − 1 represents the relative fluorescence intensity of DCF at 522 nm with a different irradiation time. [TCF and TCFBr] = 5 μmol/L.

NPs have been widely explored in PDT thanks to their long-term blood circulation and tumor accumulations [33]. The synthesized PSs and dextran modified with CHS were self-assembled into NPs by the nanoprecipitation method [6]. The PS entrapment ratios of dextran-CHS to TCF and TCFBr were calculated to be 61% and 81.3%, respectively. The particle sizes of TCF NPs and TCFBr NPs determined by DLS were 116 nm and 122 nm, respectively (Figure S3). The diameter of TCFBr NPs barely changed in different medium within 3 days and scarcely altered in water within 7 days (Figures S4 and S17), indicating good stability. Under the same molarity and light conditions, PSs NPs are forecast to decompose ABDA more efficiently than PSs. In fact, TCF NPs and TCFBr NPs decomposed 20.7% and 41.2% of ABDA within 5 min illumination, respectively (Figure 3C). The ^1^O_2_ generation efficiency of TCFBr NPs is 1.26 times that of TCFBr and 1.99 times that of TCF NPs, in line with our expectation that PSs NPs doped with bromine atoms produced more ^1^O_2_ than those without encapsulation or bromine atoms.

As shown in Figure S2, the fluorescence intensity fluctuations represent the amount of DCF generated by ROS oxidization of DCFH, and it is obvious that TCFBr NPs produce more ROS than TCF NPs. By comparing the changes of fluorescence intensity at 522 nm, we found that TCFBr NPs generated 2.03 times ROS than TCFBr (Figure 3D). The infrared thermal camera monitored the insignificant temperature changes of NPs in PBS continuously irradiated with a 530 nm laser for 20 min in real time was noted, meaning that TCFBr NPs had no photothermal effect under the treatment condition (Figure S5).

### 2.3. Selective Photodynamic Therapy of Cancer Cells with Dextran-CHS Encapsuled PSs

#### 2.3.1. Intracellular ROS

**Figure 4:**
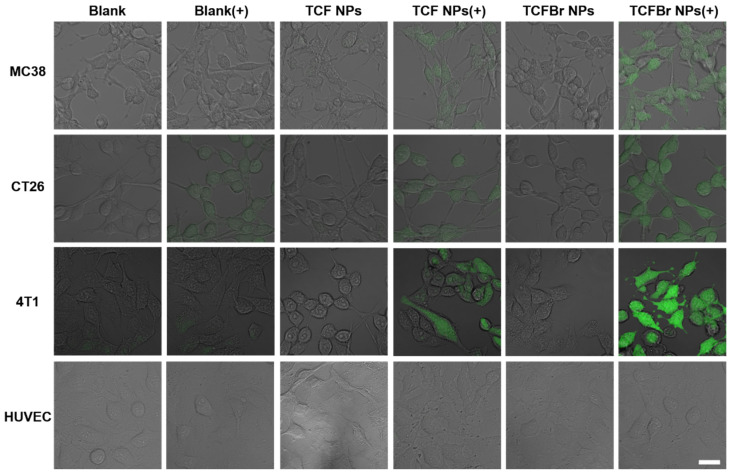
Intracellular ROS evaluated by DCFH-DA. CLSM images of MC38 cells, CT26 cells, 4T1 cells, and HUVEC cells incubated with DCFH-DA after treatment with PBS, TCF NPs, or TCFBr NPs in dark or upon light irradiation (200 mW/cm^2^) for 2 min. [TCF NPs and TCFBr NPs] = 12.5 μmol/L. (+) represents laser irradiation. Scale bars = 25 μm.

ROS can damage cells through programmed or physiological mechanisms for cell death [34]. To confirm that PSs NPs can selectively target cancer cells and generate ROS to induce cancer cell death, we monitored the status of intracellular ROS and levels of live/dead staining in several different types of cells treated with PSs NPs by confocal laser scanning microscopy (CLSM). 2′,7′-Dichlorofluorescent indigoacetate (DCFH-DA) that can be deacetylated to DCFH by cellular esterases and subsequently oxidized by ROS to green fluorescent DCF was employed to estimate intracellular ROS. Similar to the blank group, the cells in the dark group did not exhibit the existence of DCF that can emit green fluorescence, indicating that the photodynamic reaction process was not initiated in the absence of light (Figure 4). Murine colon cancer (MC38) cells and murine colon tumor (CT26) cells are both mouse colon cancer cells. It can be observed that colon cancer cells treated with TCFBr NPs plus irradiation stimulated much brighter green fluorescence than other groups, including TCF NPs plus irradiation, and so did the murine breast cancer (4T1) cells. These results show that TCFBr NPs produced more ROS in cancer cells than TCF NPs, which was consistent with the previous test results in solution and further illustrated that simply adding bromine atoms can reinforce ROS generation ability. In addition, HUVEC cells treated with PSs covered by dextran-CHS scarcely let out green fluorescence under the same treatment circumstances, which may be primarily attributed to the limited uptake of dextran-CHS encased PSs by HUVEC cells, suggesting that this method is promising to lessen the toxicity to normal cells.

#### 2.3.2. Live/Dead Staining

**Figure 5:**
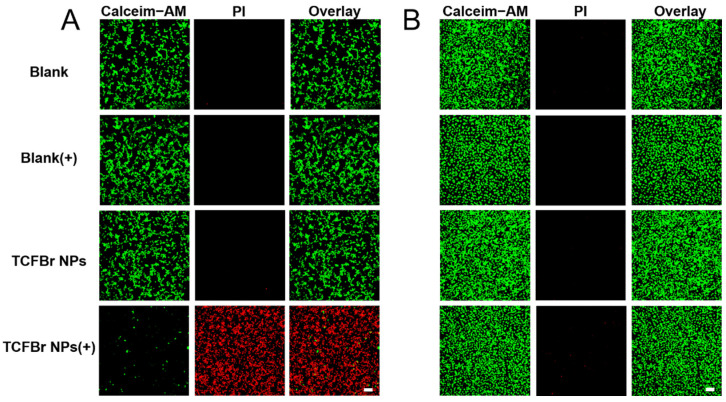
CLSM images of CT26 cells (**A**) and HUVEC cells (**B**) by live-dead cell staining assay after treatments with PBS, TCF NPs, or TCFBr NPs in dark or upon green light irradiation (200 mW/cm^2^) for 20 min. [TCF NPs and TCFBr NPs] = 12.5 μmol/L. (+) represents laser irradiation. Scale bars = 100 μm.

The phototoxicity and selective killing of TCFBr NPs were also appraised by live/dead staining approach using Calcein acetoxymethyl ester/propidium iodide (Calcein-AM/PI) to manifest living (green) and dead (red) cells. Calcein-AM is a fluorescent dye that can penetrate cells to test cell viability. It can smoothly enter intact living cells and then be degraded by non-specific esterase existing in living cells to create the bright green fluorescence signal when excited by a 488 nm laser, and its intensity is proportional to the esterase activity in cells. As esterase activity is associated with the cellular condition, the cell vitality can be inferred from the green fluorescence intensity. PI is a common fluorescence nuclear dye that can simply infiltrate destroyed cells and then embed in double-stranded DNA to emit red fluorescence when excited by a 561 nm laser [35]. As the fluorescent signal was represented in Figure 5, CT26 cells and HUVEC cells showed brilliant green fluorescence after incubating with TCFBr NPs in darkness for 24 h. Apparently, MC38 cells and 4T1 cells exposed to light or treated with TCFBr NPs in dark also showed maximum green fluorescence and minimum red fluorescence (Figure S6). Consequently, TCFBr NPs showed biocompatibility for hardly exhibited cytotoxicity without irradiation.

The difference in fluorescence proportion between CT26 cells and HUVEC cells both treated with TCFBr NPs upon 530 nm irradiation can be distinguished, revealing that TCFBr NPs could selectively damage CT26 cells while causing negligible cytotoxicity to HUVEC cells, even though there was exposure to light (Figure 5). Likewise, under light irradiation, red fluorescence that was significantly brighter than green fluorescence was also noticed in MC38 cells and 4T1 cells handled with TCFBr NPs, demonstrating that TCFBr NPs plus irradiation performed an overwhelming PDT killing capacity in comparison to other groups and exerted a satisfactory phototherapeutic effect on MC38 cells and 4T1 cells (Figure S6).

#### 2.3.3. MTT

**Figure 6:**
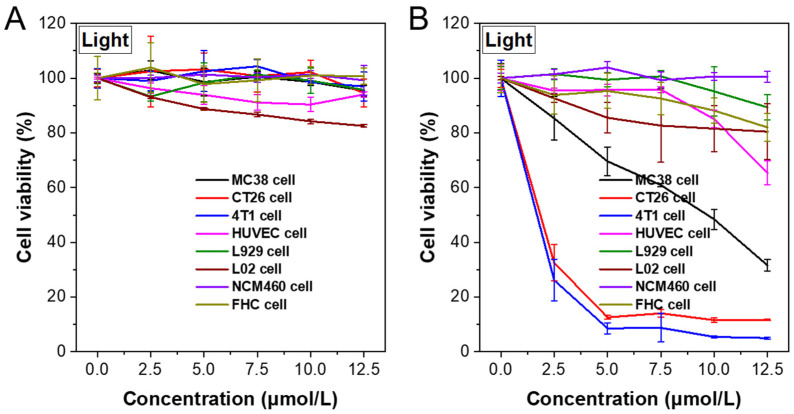
MC38, CT26, 4T1, HUVEC, L929, L02, NCM460, and FHC cell viability with different concentrations of TCFBr NPs in darkness (**A**) or upon light irradiation (**B**). The light power intensity is 200 mW/cm^2^ (20 min).

Afterward, the PDT efficiency of TCFBr NPs with gradient concentrations was quantified by 3-(4,5-dimethylthiazol-2-yl)-2,5-diphenyltetrazolium bromide (MTT) assay. Even after being incubated with higher concentrations of TCFBr NPs in the dark for 24 h, these several types of cells still maintained relatively high cell viability. The cell viability decreased slightly in the absence of light, which highlighted the fact that TCFBr NPs defeated cells through light-dependent PDT and supported the comment that TCFBr was a reliable PS with negligible dark toxicity (Figure 6A). Murine fibroblast (L929) cells and human liver (L02) cells are normal cells. Both normal intestinal epithelial (NCM460) cells and fetal human colon (FHC) cells are human normal colon cells. After 20 min of green light irradiation, the cell viability of MC38 cells, CT26 cells, 4T1 cells, HUVEC cells, L929 cells, L02 cells, NCM460 cells, and FHC cells incubated with 10 μmol/L TCFBr NPs was 48.4%, 11.6%, 5.4%, 85.0%, 85.8%, 81.5%, 100.6%, 88.2%, respectively (Figure 6B), which proved that TCFBr NPs possessed photodynamic ablation effects on colon cancer cells and relatively low destruction to normal cells.

## 3. Materials and Methods

Preparation of dextran-CHS. First, 200 mg dextran (80,000 Da) was dissolved in 10 mL DMSO, and then CHS (121.6 mg, 0.2 mmol), 4-dimethylaminopyridine (36.7 mg, 0.3 mmol), and EDC.HCl (57.5 mg, 0.3 mmol) was dissolved in 3 mL DMSO and stirred at room temperature for 1 h to activate the carboxyl group. The activated reaction solution was added to the dextran solution drop-by-drop and stirred at room temperature for 48 h. After the reaction stopped, 52 mL anhydrous ethanol was added to precipitate white products, which were filtered and freeze-dried to collect dextran-CHS stored in the refrigerator at 4 °C.

Preparation of polysaccharide NPs. First, 5 mg dextran-CHS was dissolved in 5 mL water by stirring ultrasound. A total of 1 mg TCF NPs or TCFBr NPs dissolved in 0.5 mL DMSO was added drop-by-drop to the dextran-CHS solution, and then the mixed solution was stirred at room temperature for 24 h and centrifugated at 800 rpm for 5 min. Finally, the dextran-CHS@TCF or dextran-CHS@TCFBr was transferred to a dialysis bag (MW 12,000–14,000), placed in pure water for 48 h, and filtered with a 0.45 μm filter to purify.

Critical micelle concentration (CMC). First, 10 mg dextran-CHS was dissolved in 125 μL DMSO by stirring ultrasound and then diluted in water with a series of concentrations. The particle-derived count rate affected by micelle formation was measured with DLS and plotted as a graph. The CMC value was the inflection point calculated by the straight lines of the multipoint linear simulation.

^1^O_2_ generation detection. ABDA (50 μmol/L) was mixed with PSs (TCF or TCFBr, 5 μmol/L) in water solution (1 mL), and irradiated by green light (60 mW/cm^2^). The absorption spectra of the mixture were measured every minute.

Total ROS measurements by fluorescence analysis. The general ROS generation detections were performed using DCFH as the indicator. DCFH (0.15 μg/mL) and PSs (TCF or TCFBr, 5 μmol/L) were mixed in an aqueous solution (1 mL) and irradiated by green light (60 mW/cm^2^). Changes in fluorescence intensity at 522 nm were monitored to indicate the rate of ROS generation.

Intracellular ROS detection. DCFH-DA was used as an indicator to detect the generation of ROS in cells under laser irradiation. MC38 cells were seeded and cultured for 24 h. Following incubation with 12.5 μmol/L TCF NPs or TCFBr NPs for 12 h in the dark, DCFH-DA (10 μmol/L) was used to treat cells for 20 min. After that, cells were washed twice with PBS and then the cells in the light group were irradiated under a 530 nm laser (200 mW/cm^2^) for 2 min, while the cells of other groups were incubated in the dark during this process. The DCFH-DA was excited at 488 nm and the emission was collected from 500 to 550 nm. The cellular green fluorescence intensity was observed by CLSM to estimate the intracellular ROS production.

Live-dead cell staining. MC38 cells were treated with 12.5 μmol/L TCF NPs or TCFBr NPs for 12 h. Subsequently, the cells were exposed to 530 nm irradiation (200 mW/cm^2^, 20 min) or incubated in the dark. After the treatment, the cells were further cultured for 3 h. Finally, the cells were washed with PBS and stained with Calcein-AM/PI for 30 min, and the residual dyes were washed out with PBS three times. The green fluorescence of Calcein-AM and the red fluorescence of PI were observed by CLSM to evaluate the live and dead cells, respectively. Conditions: excitation wavelength: 488 nm for Calcein-AM, 561 nm for PI; emission filter: 500–550 nm for Calcein-AM, 570–616 nm for PI.

In vitro cytotoxicity assay. For the cell viability test, ct26 cells were seeded in 96 well plates (4000 cells in 100 μL medium per well). After 12 h incubation, ct26 cells were treated with different concentrations of TCFBr NPs for 12 h and then illuminated with white light for 20 min. MTT (20 μL, 5 mg/mL) was added into the medium at 12 h post-light treatment. After 4 h, the supernatant was discarded, and each well was added to 150 µL DMSO. After being gently shaken for 10 min, the optical density (OD) of each well at 490 nm was measured by a microplate reader.

## 4. Conclusions

In summary, to meet the challenge of optimizing PDT effects for the effective and precise treatment of colon cancer cells, we brominated the PS, TCF. We then used dextran-CHS to encapsulate the halogenated PSs. With two incorporated bromine atoms, the obtained brominated TCFBr has a better ^1^O_2_ generation efficiency. Hydrophobic modification of dextran with CHS enables it to self-assemble with PSs into NPs, which are stable with particle size of approximately 120 nm, and the total ROS production of TCFBr NPs is twice that of TCF NPs in the solution. Confocal microscopy observed that TCFBr NPs with bromine atoms induced more ROS that could greatly erode cancer cells. It is proved that TCFBr combined with dextran-CHS had preeminent photoactive toxicity and permitted selectively killed cancer cells while causing less damage to normal cells at the suitable concentration in vitro tests. Based on the advantages mentioned above, it is relatively creditable that polysaccharide-encapsulated PSs are promising in colon cancer therapy.

## Data Availability

The data presented in this study are available from the corresponding author upon reasonable request.

## Supplementary Materials

The following supporting information can be downloaded at: https://www.mdpi.com/article/10.3390/molecules28114404/s1, Figure S1: Absorption spectra; Figure S2: Fluorescence spectra; Figure S3: Dynamic light scattering (DLS) size distribution of (A) TCF NPs, and (B) TCFBr NPs; Figure S4: Particle size changes of TCFBr NPs within seven consecutive days; Figure S5: Thermal effect of TCFBr NPs upon continuous laser irradiation (200 mW/cm^2^); Figure S6: CLSM of live/dead calcein AM/PI staining; Figure S7: Cell viability; Figure S8: ^1^H NMR spectrum of 1 in *d*-chloroform; Figure S9: ^13^C NMR spectrum of 1 in *d*-chloroform; Figure S10: ^1^H NMR spectrum of 2 in *d*-chloroform; Figure S11: ^13^C NMR spectrum of 2 in *d*-chloroform; Figure S12: ^1^H NMR spectrum of TCF in *d*-chloroform; Figure S13: ^13^C NMR spectrum of TCF in *d*-chloroform; Figure S14: ^1^H NMR spectrum of TCFBr in *d*-chloroform; Figure S15: ^13^C NMR spectrum of TCFBr in *d*-chloroform; Figure S16: ^1^H NMR spectrum of dextran, CHS, and dextran-CHS in *d*-chloroform; Figure S17: Stability test of (A) TCF NPs and (B) TCFBr NPs under different biological medium. Scheme S1. The synthetic routes to dextran-CHS, TCF, TCFBr.

## Sample Availability

Samples of the compounds are available from the authors.
