# Supplementary material for: Dextran-Cholesterol Carrier Encapsulated Efficient Photosensitizer for the Photodynamic Killing of Cancer Cells
Source: Molecules. 2023 May 28;28(11):4404. doi: 10.3390/molecules28114404 (PMC10254848; doi:10.3390/molecules28114404)
Supplement: Supplementary file 1 [file molecules-28-04404-s001.zip › molecules-2388382-supplementary.pdf]

Dextran−Cholesterol Carrier Encapsulated Efficient Photosensitizer for the Photodynamic Killing of Cancer Cells

Biru Wu [1], Zhuoheng Gan [1], Shengchang Tao [2], Qiang Wang [1], Yuchen Song [1], Hua Zhong [3,*] and Fang Hu [1,4,*]

[1] Biomaterials Research Center, School of Biomedical Engineering, Southern Medical University, Guangzhou 510515, China
[2] Department of Pharmacy, Affiliated Dongguan Hospital, Southern Medical University, Dongguan 523059, China
[3] Department of Orthopaedics, The Fifth Affiliated Hospital, Southern Medical University, Guangzhou 510900, China
[4] Division of Laboratory Medicine, Zhujiang Hospital, Southern Medical University, Guangzhou 510282, China
* Correspondence: zhong8099@163.com (H.Z.); hufang19@smu.edu.cn (F.H.)

Materials and Methods

Materials. (4−(diphenylamino)phenyl)boronic acid was purchased from Leyan; tris(4−bromophenyl)amine, 5−bromothiophene−2−carbaldehyde, (5−formylthiophen−2−yl)boronic acid and 4−dimethylaminopyridine were purchased from Bidepharm; tetrakis(triphenylphosphine)palladium ($Pd(pph_3)_4$) was purchased from Inochem; potassium carbonate and dextran were purchased from Aladdin; 9,10−Anthracenediyl−bis(methylene) dimalonic acid (ABDA) was purchased from Sigma; piperidine was purchased from General−reagent; cholesteryl hemisuccinate (CHS) and 2′,7′−Dichlorodihydrofluorescein (DCFH) were purchased from yuanye Bio−technology; 1−(3−Dimethylaminopropyl)−3−ethylcarbodiimide (hydrochloride) (EDC.HCl) was purchased from Macklin; All other chemicals were obtained from commercial sources and used as received without further purification. All non−aqueous reactions were carried out under a nitrogen atmosphere in oven−dried glassware. Milli−Q water was used in all the related experiments.

Phosphate buffered saline (PBS), Dulbecco's modified Eagle medium (DMEM), Roswell Park memorial institute (RPMI−1640) medium, fetal bovine serum origin (FBS), and Penicillin−Streptomycin liquid were purchased from Gibco. 2′,7′−Dichlorofluorescin diacetate (DCFH−DA), and calcein−AM/PI cell viability/cytotoxicity assay kit were purchased from Beyotime.

3−(4,5−dimethylthiazol−2−yl)−2,5−diphenyltetrazolium bromide (MTT) was purchased from Solarbio.

Instruments. NMR spectra were measured on a Bruker AscendTM 400 NMR spectrometer. The extent of reaction was monitored by thin layer chromatography (TLC) using silica gel plates with fluorescent indicators UV254 and UV365 after the plates were subjected to elution in the TLC chamber. Flash column chromatography was carried out using Rhawn silica gel (200−300 mesh). UV−vis absorption spectra were taken on a Shimadzu Model UV−2600 spectrometer. Fluorescence spectra were measured on a Shimadzu Model RF−6000 spectrofluorometer. All UV and PL spectra were collected at 24 ± 1 °C. Particle size and size distribution were determined by dynamic light scattering (DLS) with a particle size analyzer (90 Plus, Brookhaven Instruments Corporation, United States) at a fixed angle of 90° at room temperature. The power intensity of light was measured by LWP10W−A optical power meter (Beijing Laserwave OptoElectronics Technology Co., Ltd). The photodynamic therapy experiments were carried out by a LWGL530−2W green light source (Beijing Laserwave OptoElectronics Technology Co., Ltd). Cytotoxicity tests were carried out on a microplate reader (Synergy H1, BioTek) by using MTT assays. Confocal Laser Scanning microscopy images were recorded on a confocal laser scanning microscope (CLSM, Nikon−A1 HD25).

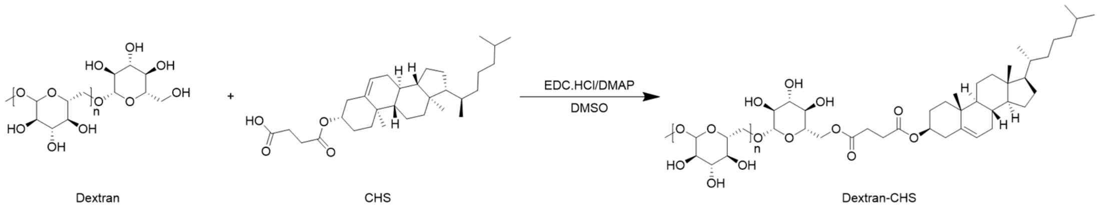

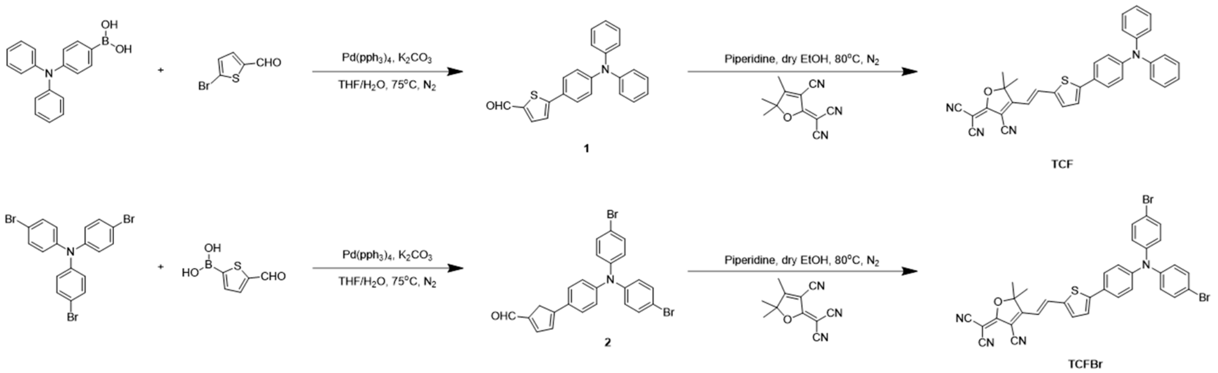

Scheme S1. The synthetic routes to dextran−CHS, TCF, TCFBr.

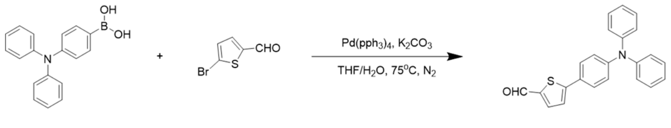

1

Synthesis of 5−(4−(diphenylamino)phenyl)thiophene−2−carbaldehyde (1). (4−(diphenylamino)phenyl)boronic acid (660 mg, 2.28 mmol), 5−bromothiophene−2−carbaldehyde (520.4 mg, 2.74 mmol), Pd(pph$_3$)$_4$ (10 mg, 0.009 mmol) were mixed and dissolved in THF (60 mL), stirred under argon atmosphere. Aqueous solution of K_2CO_3 (6 mL, 2M) was then injected. The mixture continued to stir and heat at 75 ℃ for 21h. After cooling to room temperature, the reaction was quenched with water and ethyl acetate, then washed with water (20 mL × 3). The organic phase was dried with Na_2SO_4 and the solvent was removed under reduced pressure. The obtained residue was purified with chromatography (petroleum ether/ dichloromethane = 2/1, v/v) to give compound 1 as a yellow solid (549.2 mg, 67.75%). ^{1}H−NMR (400 MHz, $CDCl_3$) δ 9.85 (s, 1H), 7.71 (d, J = 4.0 Hz, 1H), 7.52 (d, J = 8.7 Hz, 2H), 7.30 (m, 5H), 7.15 – 7.05 (m, 8H). ^{13}C− NMR (100 MHz, $CDCl_3$) δ 182.53, 154.53, 149.10, 146.93, 141.29, 137.64, 129.44, 127.20, 126.10, 125.13, 123.83, 122.81, 122.32.

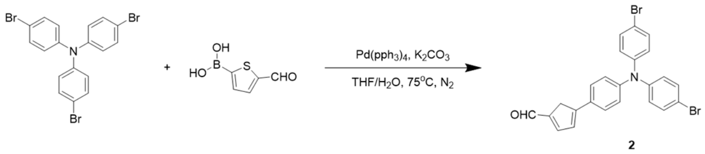

2

Synthesis of 4−(4−(bis(4−bromophenyl)amino)phenyl)cyclopenta−1,3−diene−1−carbaldehyde (2). Tris(4−bromophenyl)amine (2 g, 4.18 mmol), (5−formylthiophen−2−yl)boronic acid (2.35 g, 15.04 mmol), Pd(pph$_3$)$_4$ (18.8 mg, 0.017 mmol) were mixed and dissolved in THF (83.5 mL), stirred under argon atmosphere. Aqueous solution of K_2CO_3 (8.35 mL, 2M) was then injected. The mixture continued to stir and heat at 75 ℃ for 24h. After cooling to room temperature, the reaction was quenched with water and ethyl acetate, then washed with water (20 mL × 3). The organic phase was dried with Na_2SO_4 and the solvent was removed under reduced pressure. The obtained residue was purified with chromatography (petroleum ether/ ethyl acetate/ dichloromethane = 25/1/5, v/v/v) to give compound 2 as a yellow solid (422.7 mg, 20.53%). ^{1}H−NMR (400 MHz,

CDCl3) δ 9.87 (s, 1H), 7.72 (d, J = 3.8 Hz, 1H), 7.54 (d, J = 8.5 Hz, 2H), 7.39 (d, J = 8.6 Hz, 4H), 7.32 (d, J = 3.8 Hz, 1H), 7.02 (m, 6H). ^{13}C NMR (100 MHz, CDCl3) δ 182.61, 153.88, 148.03, 145.70, 141.71, 137.60, 132.61, 127.46, 127.37, 126.23, 123.25, 123.20, 116.64.

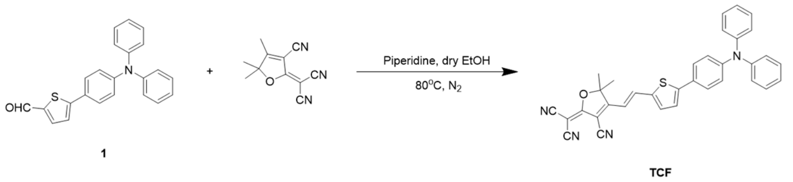

Synthesis of TCF. A solution of compound 1 (150 mg, 0.45 mmol) and 2−(3−cyano−4,5,5−trimethylfuran−2−ylidene)propanedinitrile (89.1 mg, 0.45 mmol) was refluxed in dry ethanol catalyzed by a few drops of piperidine for 19 h under nitrogen. After cooling to room temperature, the solvent was evaporated under reduced pressure. The residue was purified by silica gel chromatography eluting with petroleum ether/ dichloromethane (v/v = 1:2) to give the purple−black solid TCF (152.7 mg, 67.4%). ^{1}H−NMR (400 MHz, CDCl3): δ 7.80 (d, J = 15.8 Hz, 1H), 7.50 (d, J = 8.7 Hz, 2H), 7.44 (d, J = 4.0 Hz, 1H), 7.36 – 7.27 (m, 5H), 7.13 (m, 6H), 7.06 (d, J = 8.7 Hz, 2H), 6.62 (d, J = 15.8 Hz, 1H), 1.76 (s, 6H). ^{13}C NMR (101 MHz, CDCl3) δ 175.58, 173.00, 153.89, 149.53, 146.66, 139.55, 137.86, 137.37, 129.53, 127.15, 125.40, 124.21, 124.02, 121.85, 111.96, 111.23, 110.80, 96.99, 96.70, 29.65, 26.48.

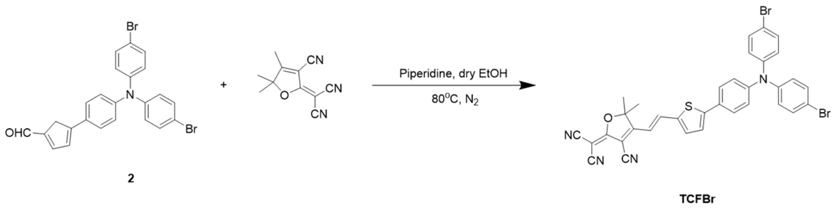

Synthesis of TCFBr. A solution of compound 2 (170 mg, 0.34 mmol) and 2−(3−cyano−4,5,5−trimethylfuran−2−ylidene)propanedinitrile (68.7 mg, 0.34 mmol) was refluxed in dry ethanol catalyzed by a few drops of piperidine for 19 h under nitrogen. After cooling to room temperature, the solvent was evaporated under reduced pressure. The residue was purified by silica gel chromatography eluting with petroleum ether/ dichloromethane (v/v = 1:2) to give the purple−black solid TCF (148.2 mg, 62.1%). ^{1}H−NMR (400 MHz, CDCl3): δ 7.80 (d, J = 15.9 Hz, 1H), 7.52 (d, J = 8.6 Hz, 2H), 7.43 (m, 4H), 7.34 – 7.26 (m, 3H), 7.03 (m, 5H), 6.65 (d, J = 15.8 Hz, 1H), 1.80

– 1.68 (m, 6H). ^{13}C NMR (101 MHz, $CDCl_3$) δ 175.48, 172.99, 152.96, 148.40, 145.49, 139.41, 138.28, 137.13, 132.70, 127.34, 126.74, 126.45, 124.43, 122.85, 117.02, 112.36, 111.91, 111.15, 110.70, 97.20, 97.07, 29.64, 26.47.

Evaluation of drug encapsulation rate. TCF and TCFBr levels were quantified by UV detection at 575 nm and 565 nm, respectively. The standard curve of drug was calculated by measuring the absorbance of different concentrations of drugs with UV−Vis. The standard curve of drug in nanoparticles was obtained under identical conditions. The amount of drug in nanoparticles was determined from the slope correlated with the standard curve. The entrapment efficiency of drug was calculated using the following equation:

$$\text{Drug entrapment (\%)} = \frac{\text{amount of drug in nanoparticles}}{\text{total amount of drug}} \times 100$$

Cell cultures. The murine colon (mc38) cells were cultured in RPMI−1640 (containing 10% heat−inactivated fetal bovine serum) at 37 °C in a humidified incubator with 5% CO2. The murine breast cancer ($4T_1$) cells, murine colon tumor (ct26) cells, human umbilical vein endothelial cells (HUVEC), murine fibroblast (L929) cells, human liver (L02) cells, normal intestinal epithelial (NCM460) cells, fetal human colon (FHC) cells were maintained in DMEM supplemented with 10% FBS, 1% streptomycin/penicillin and maintained at an atmosphere of 5% CO_2 and 95% humidified air at 37 °C. Before the experiments, the cells were pre−cultured until confluence was reached.

Photothermal properties of TCFBr NPs. The photothermal effect in TCFBr NPs solution was evaluated by an infrared thermal imager. The PBS solution of TCFBr NPs (12.5 μmol/L) was continuously exposed to a 530 nm laser (200 mW/cm2) for 20 min and the temperature was measured every min.

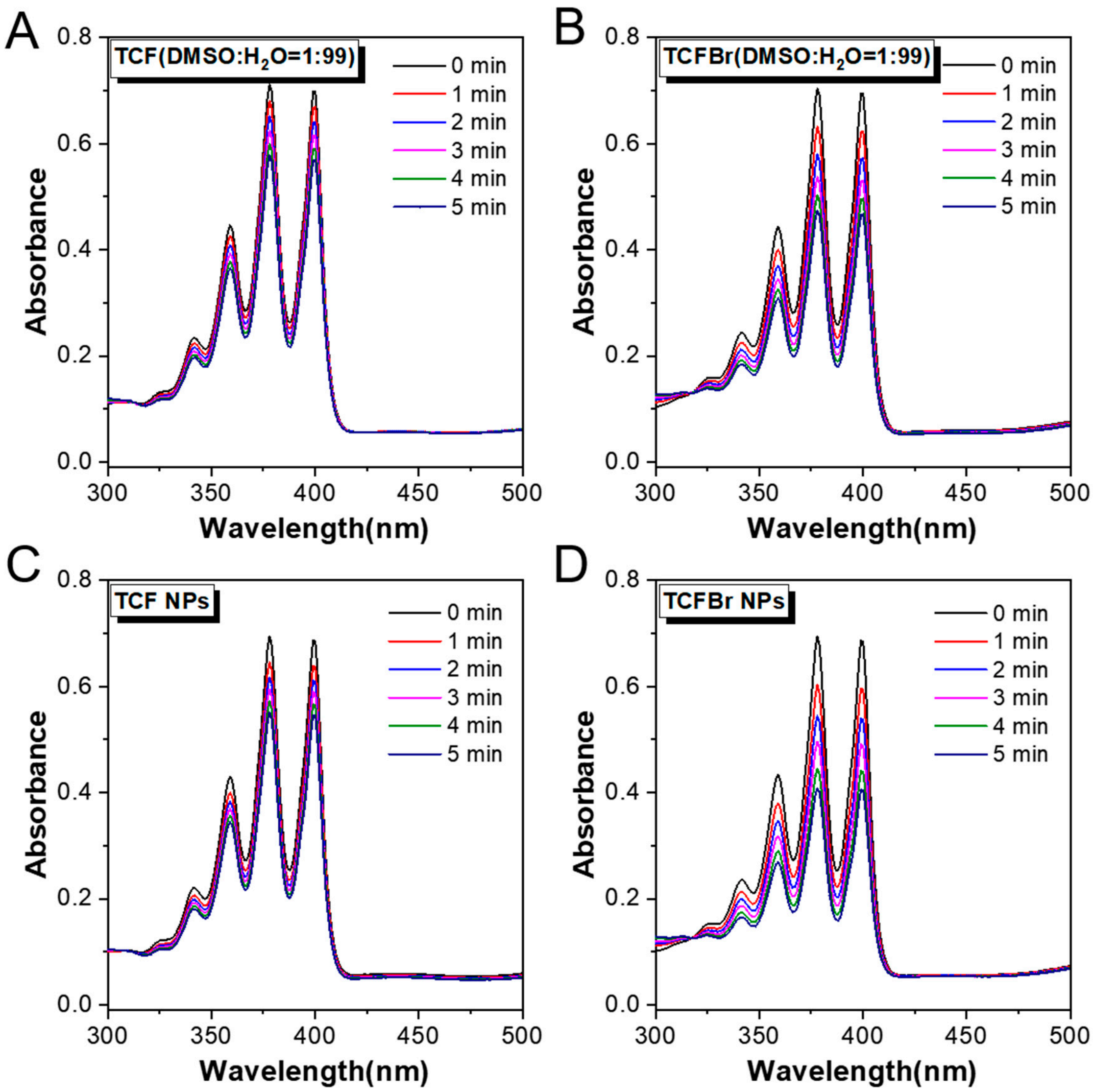

Figure S1. Absorption spectra of mixed aqueous solutions of ABDA (50 μM) with (A) TCF (DMSO: water = 1:99), (B) TCFBr (DMSO: water = 1:99), (C) TCF NPs, and with (D) TCFBr NPs (5 μmol/L) under 60 mW/cm^2 green irradiation at different times.

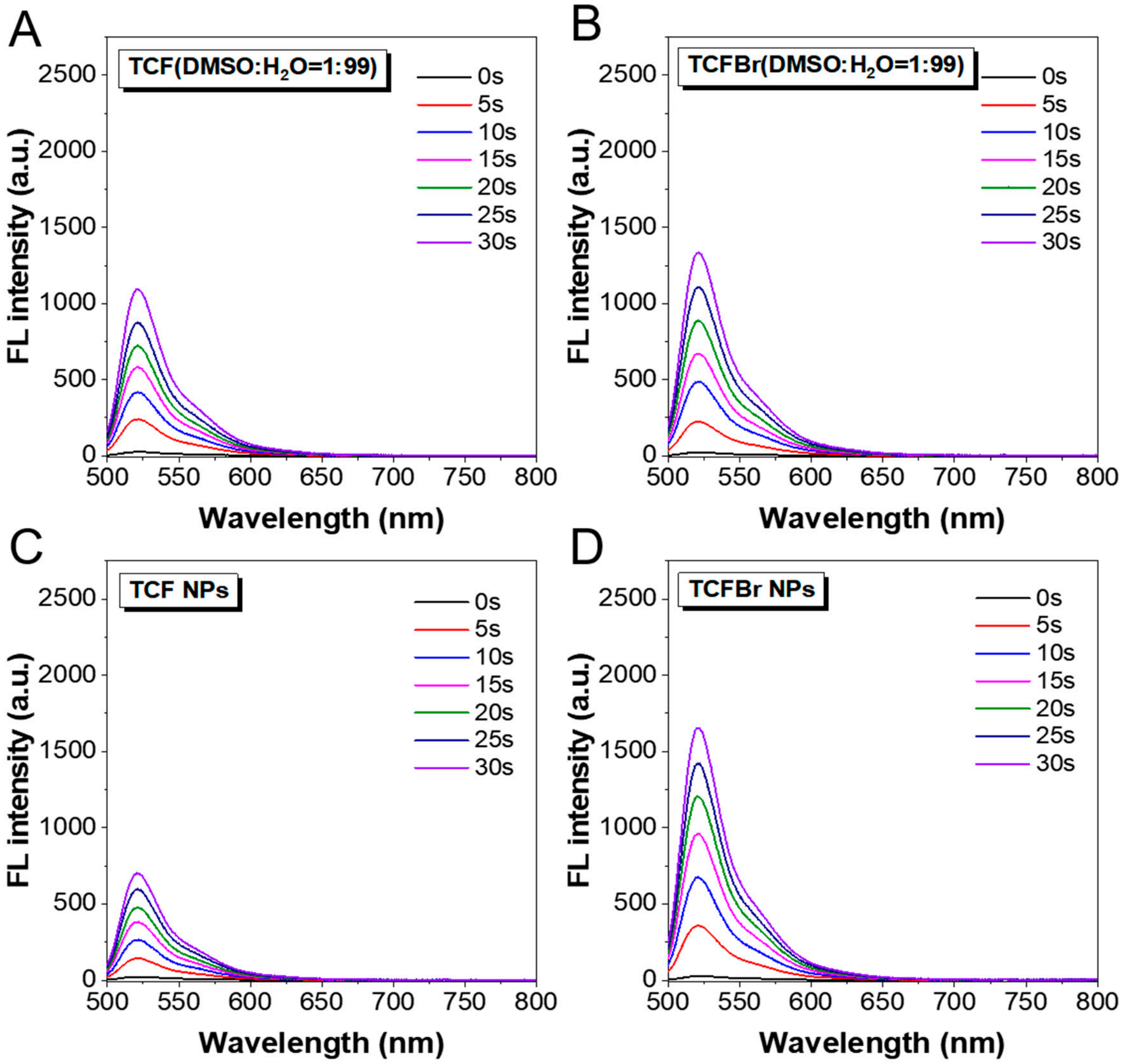

Figure S2. Fluorescence spectra of mixed aqueous solutions of DCFH (0.15 μg/mL) with (A) TCF (DMSO: water = 1:99), (B) TCFBr (DMSO: water = 1:99), (C) TCF NPs, and with (D) TCFBr NPs (5 μmol/L) with different green light irradiation times.

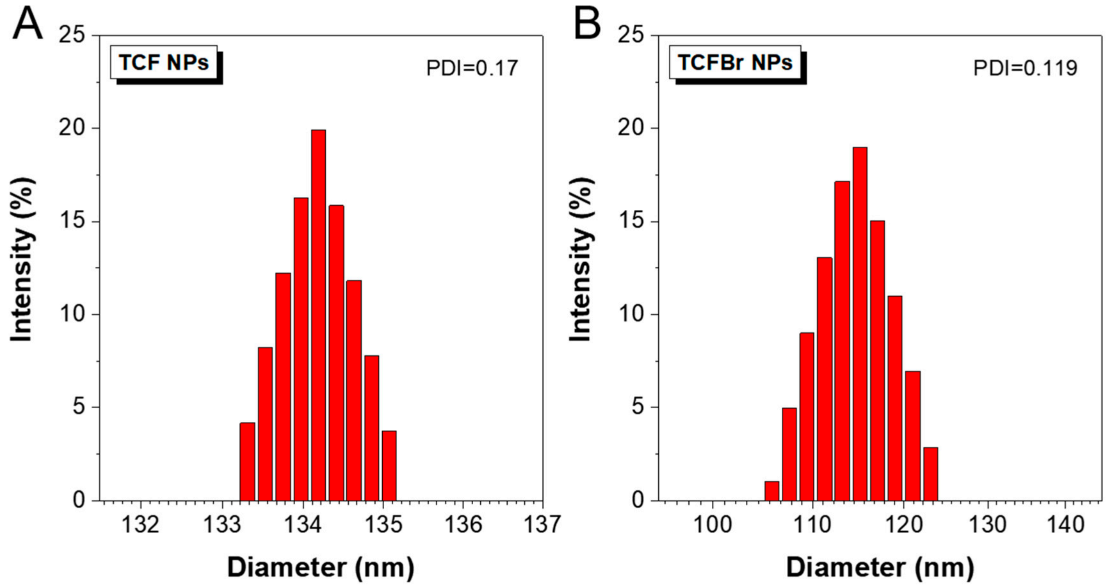

Figure S3. Dynamic light scattering (DLS) size distribution of (A) TCF NPs, and (B) TCFBr NPs.

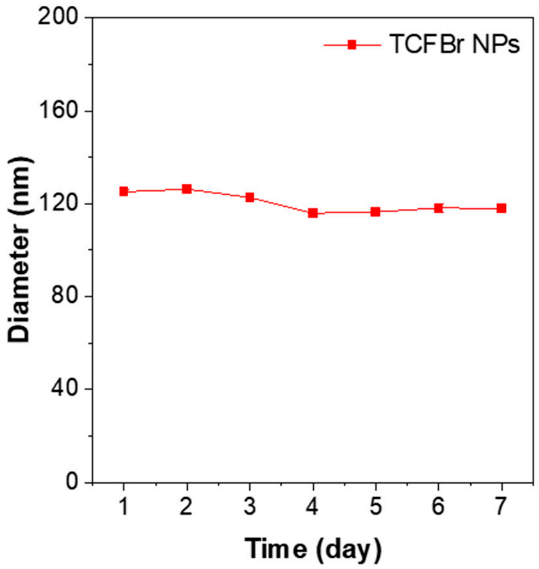

Figure S4. Particle size changes of TCFBr NPs in water within seven consecutive days.

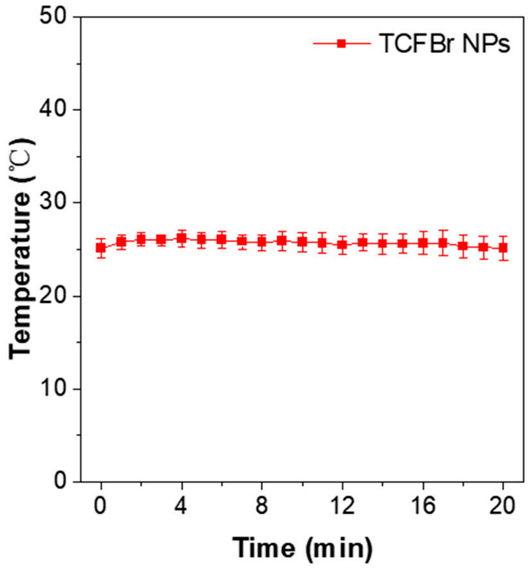

Figure S5. Thermal effect of TCFBr NPs upon continuous laser irradiation (200 mW/cm^2).

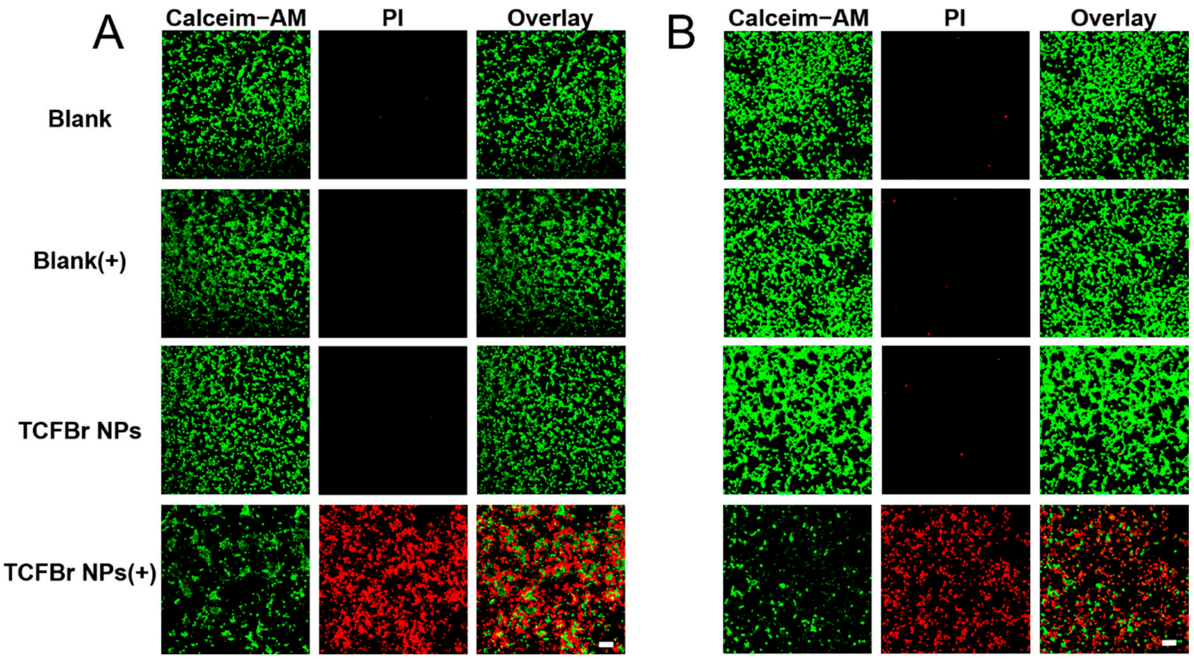

Figure S6. CLSM of live/dead calcein AM/PI staining of (A) MC38 cells, and (B) 4T1 cells treated with Blank, Blank (+), TCF NPs, and TCFBr NPs (+) for 12 h. (+) represents laser irradiation. Scale bars = 100 μm.

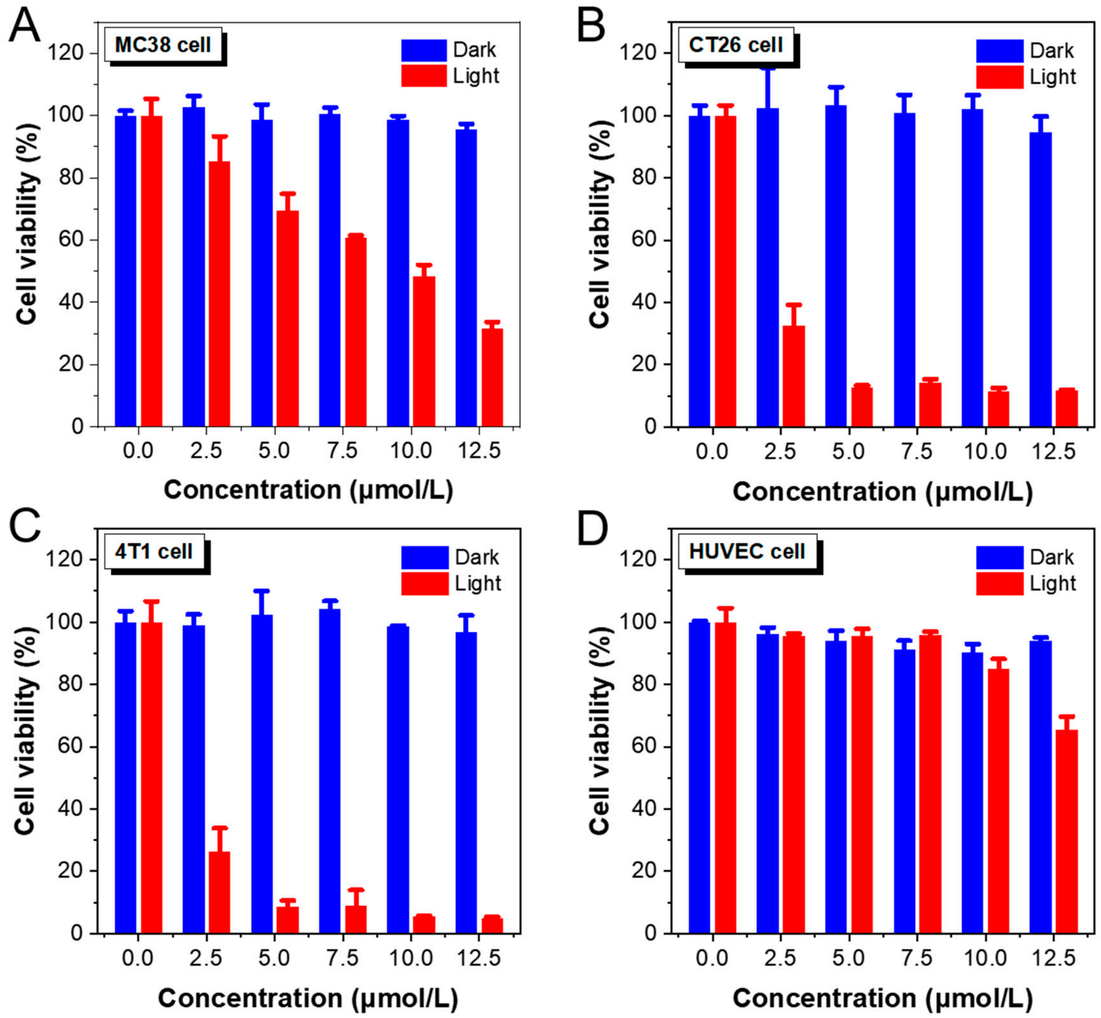

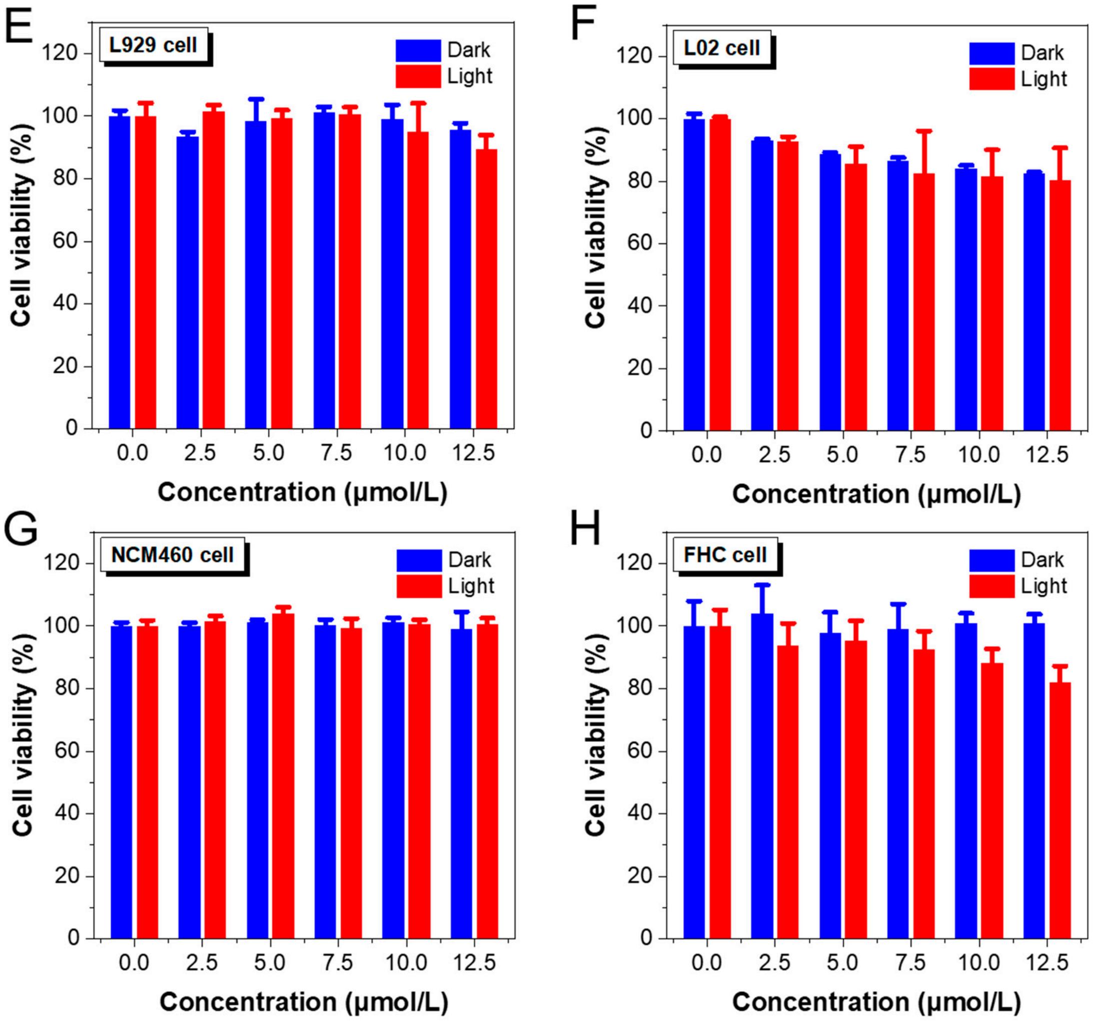

Figure S7. Cell viability of (A) MC38 cells, (B) CT26 cells, (C) 4T1 cells, (D) HUVEC cells (E) L929 cells, (F) L02 cells, (G) NCM460 cells, and (H) FHC cells upon treatment with different concentrations of TCFBr NPs in darkness or upon light irradiation (200 mW/cm^2) for 20 min.

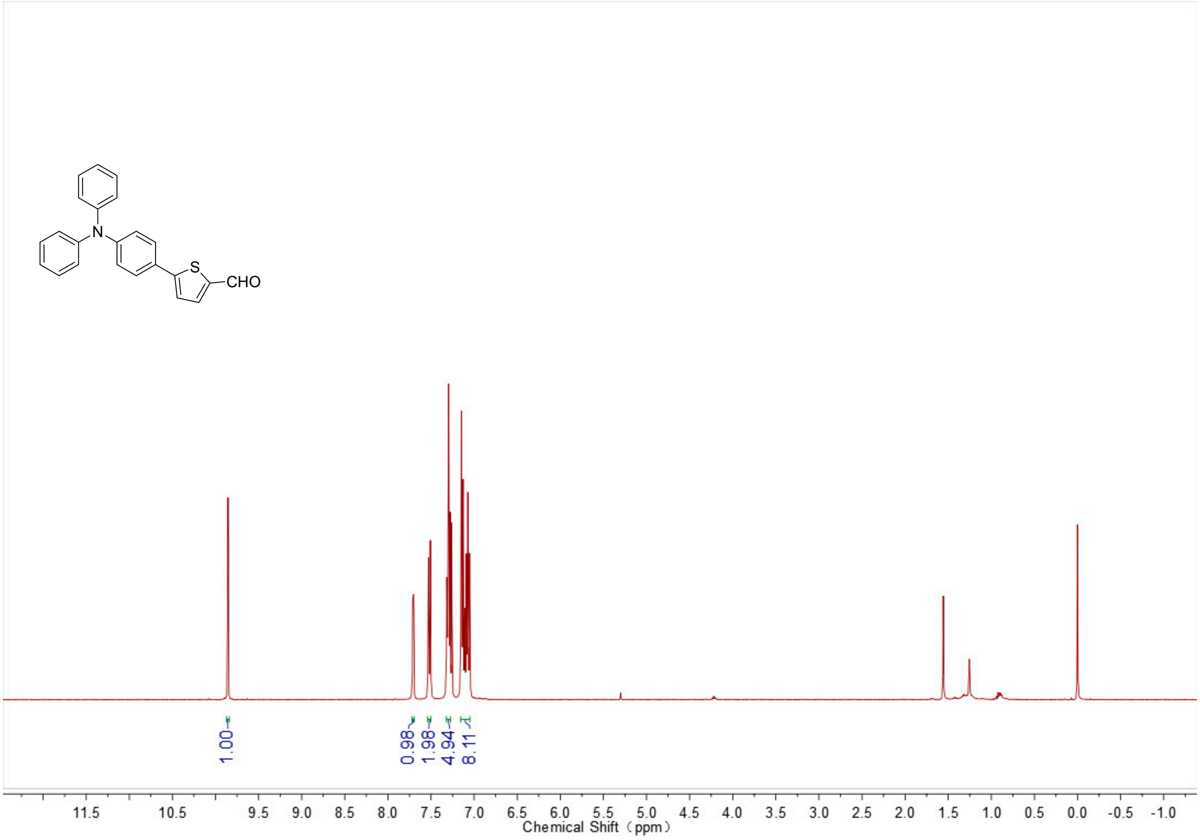

Figure S8. ^{1}H NMR spectrum of **1** in *d*−chloroform.

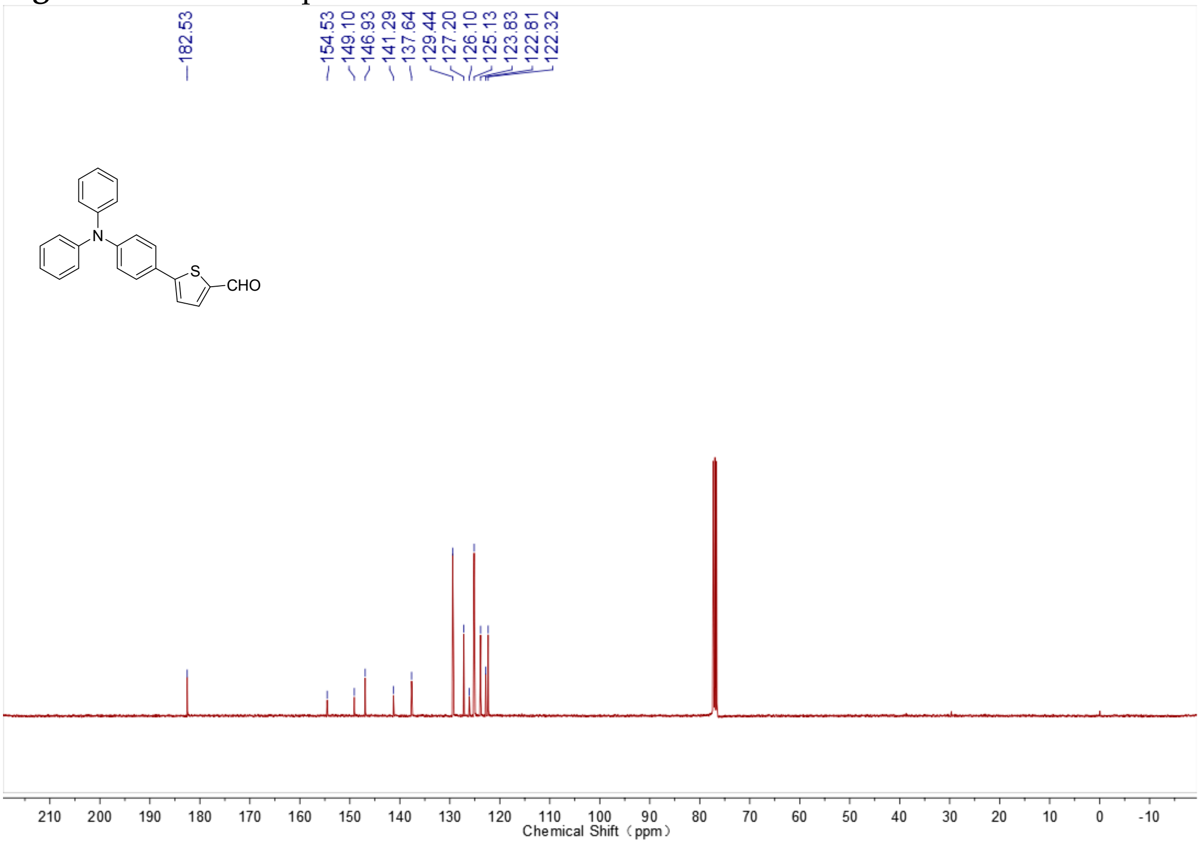

Figure S9. ^{13}C NMR spectrum of **1** in *d*−chloroform.

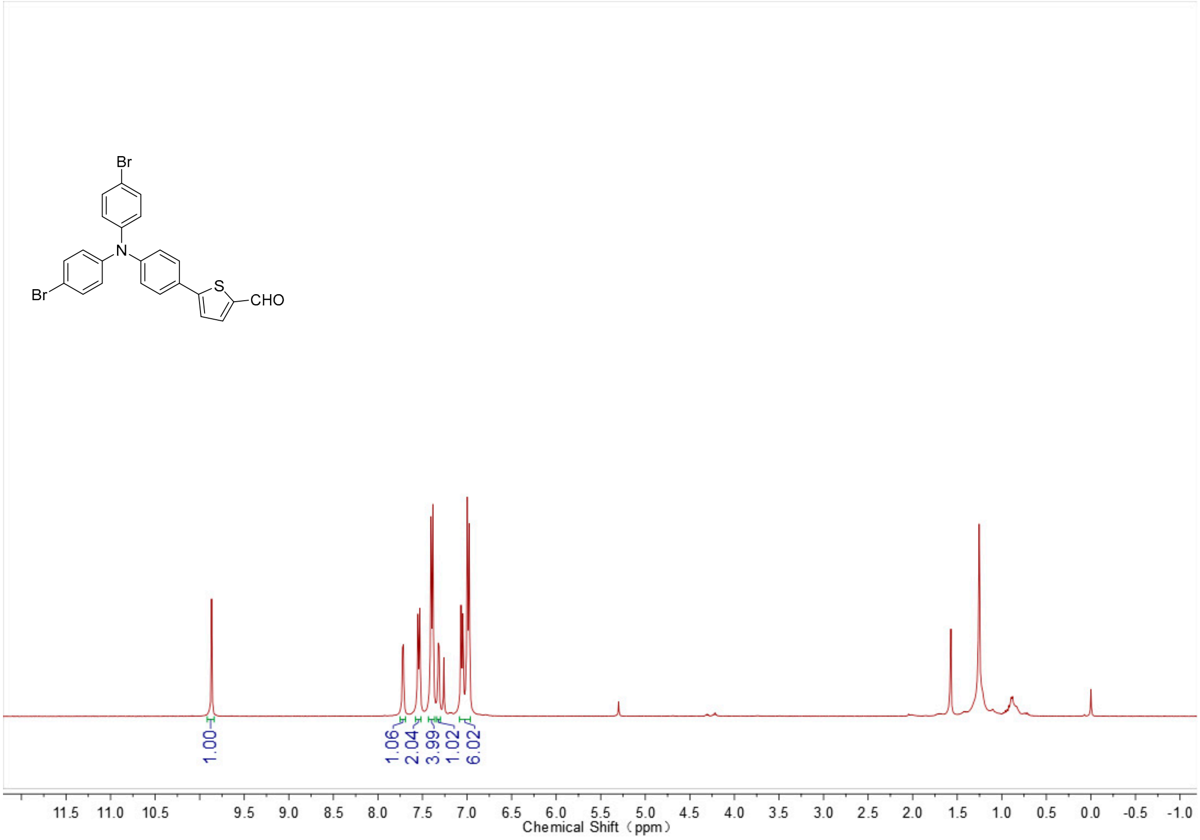

Figure S10. ^{1}H NMR spectrum of **2** in *d*–chloroform.

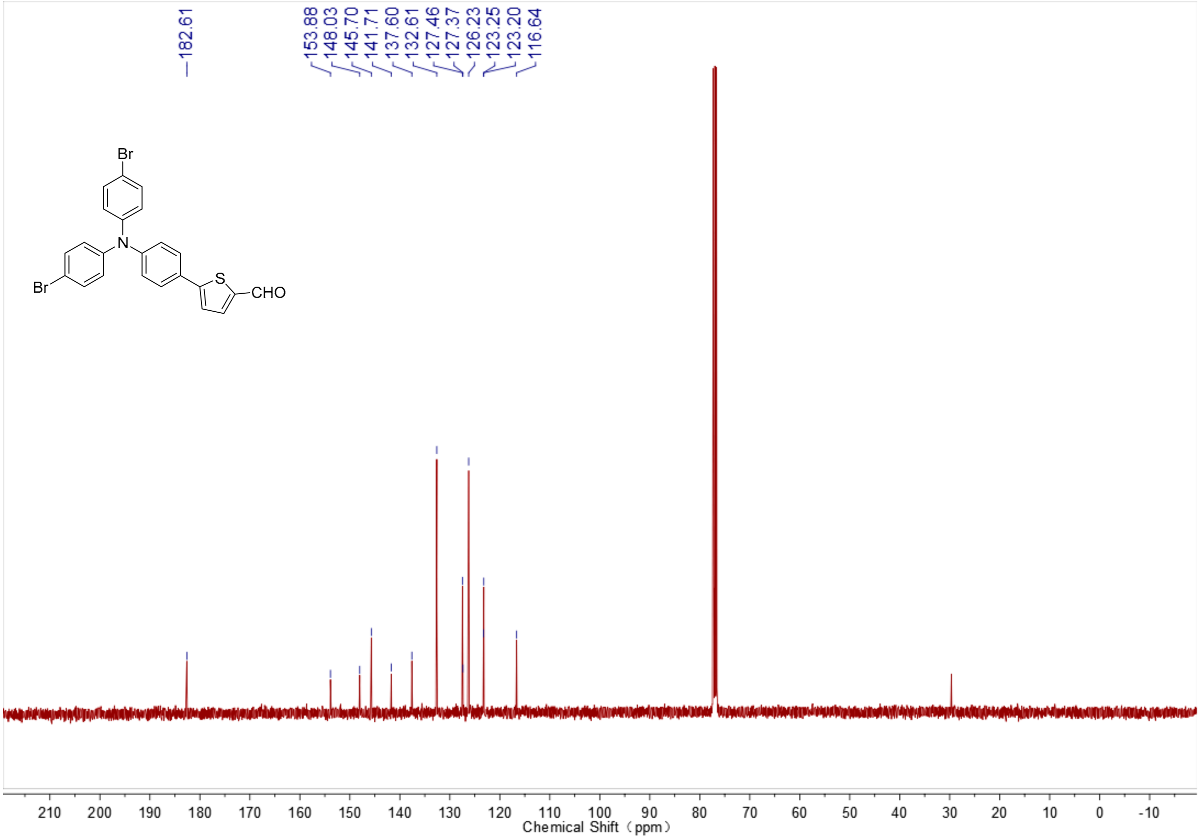

Figure S11. ^{13}C NMR spectrum of **2** in *d*–chloroform.

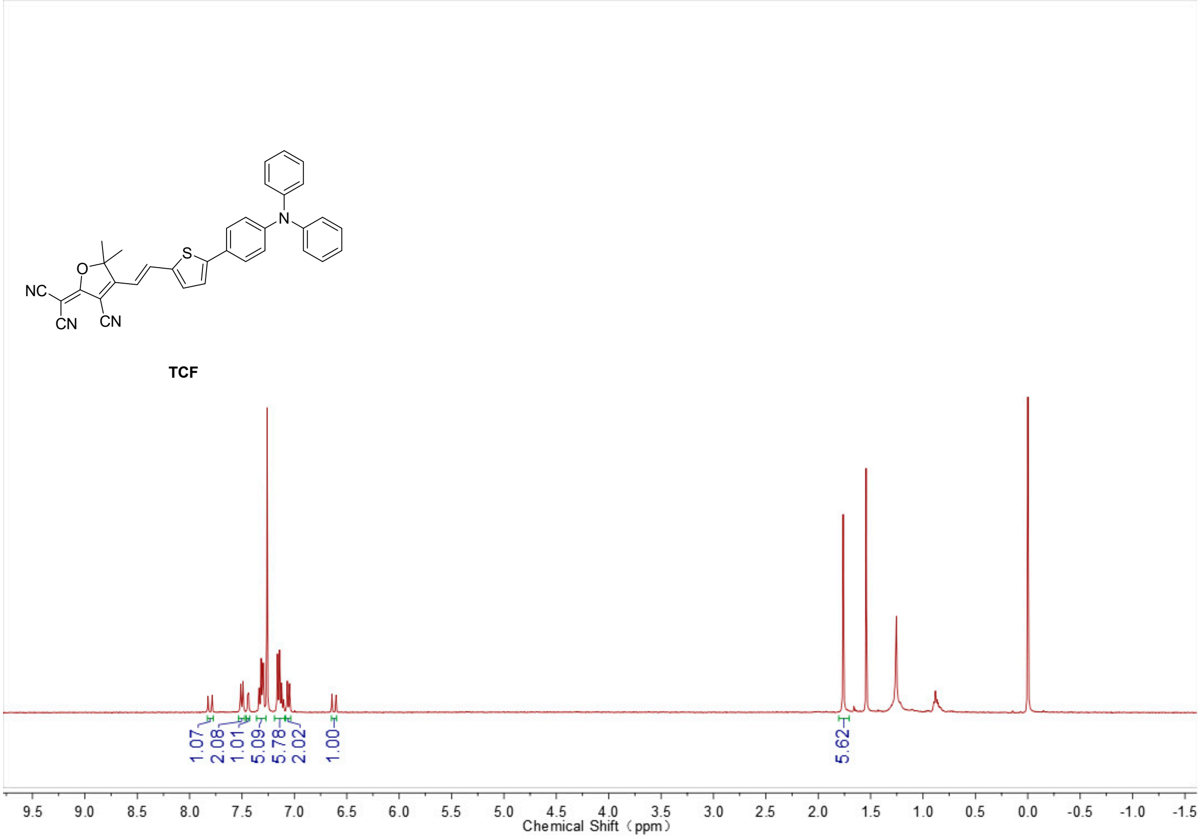

Figure S12. ^{1}H NMR spectrum of TCF in *d*−chloroform.

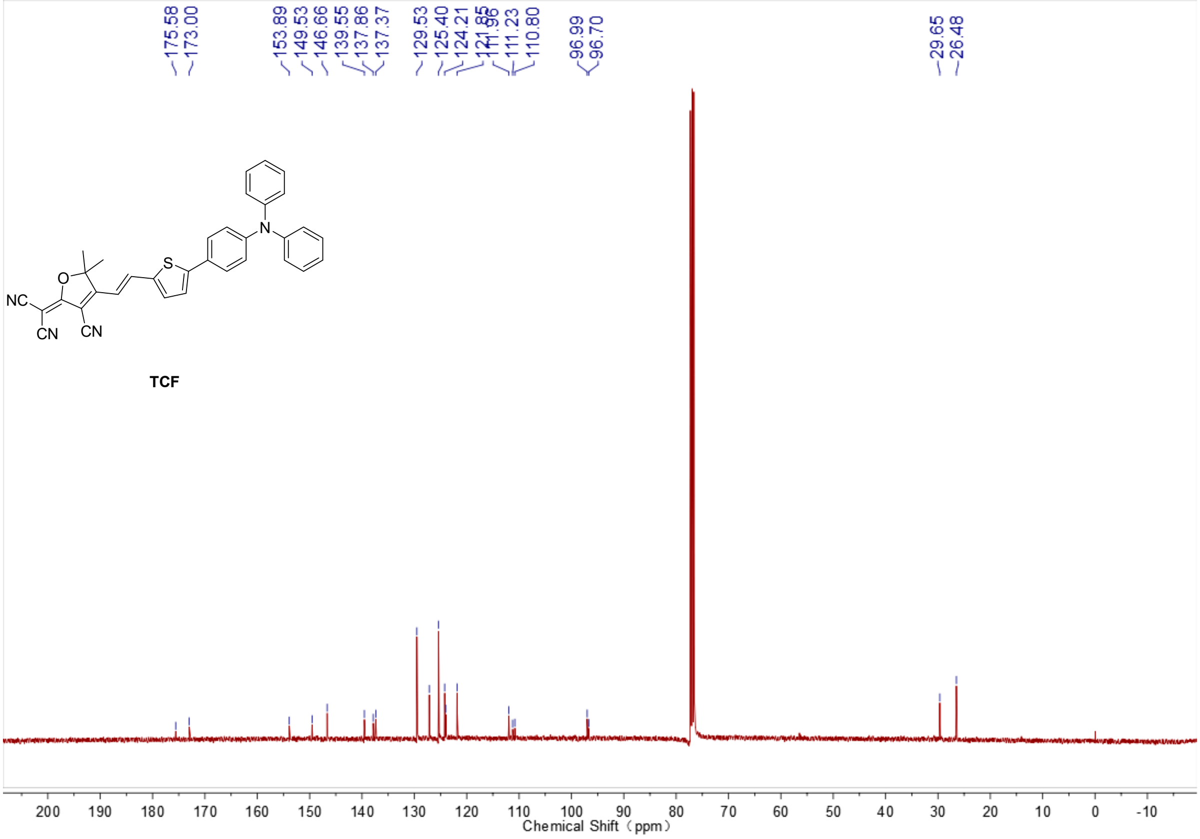

Figure S13. ^{13}C NMR spectrum of TCF in *d*−chloroform.

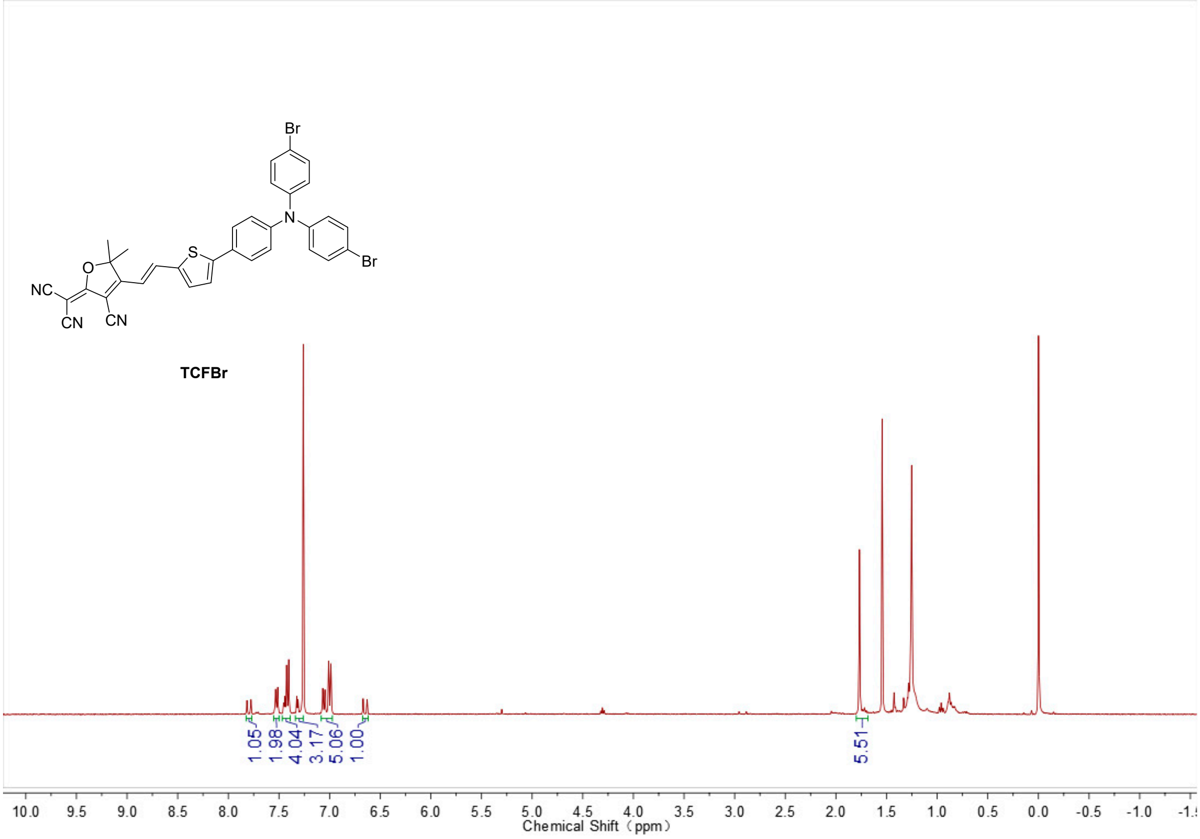

Figure S14. ^{1}H NMR spectrum of TCFBr in *d*−chloroform.

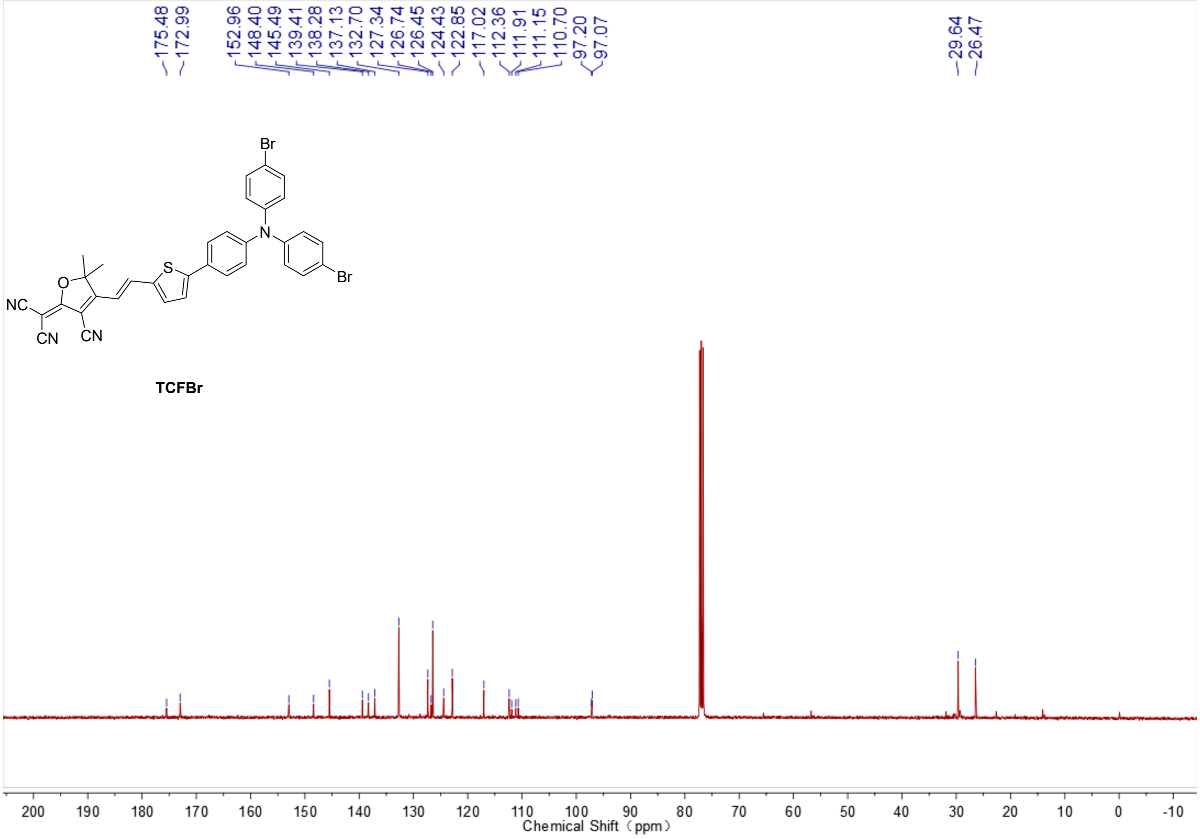

Figure S15. ^{13}C NMR spectrum of TCFBr in *d*−chloroform.

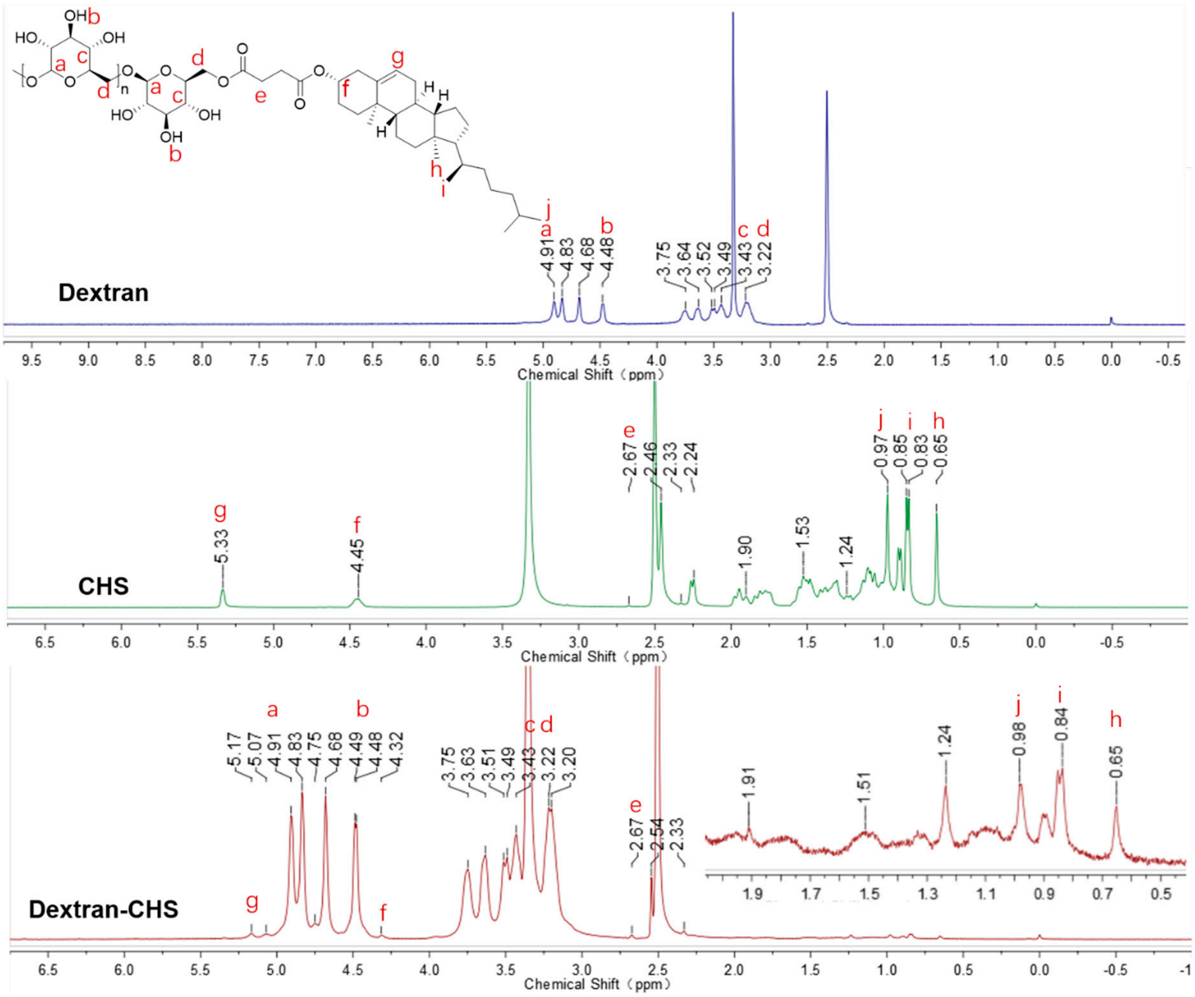

Figure S16. ^{1}H NMR spectrum of dextran, CHS, and dextran−CHS in *d*−chloroform.

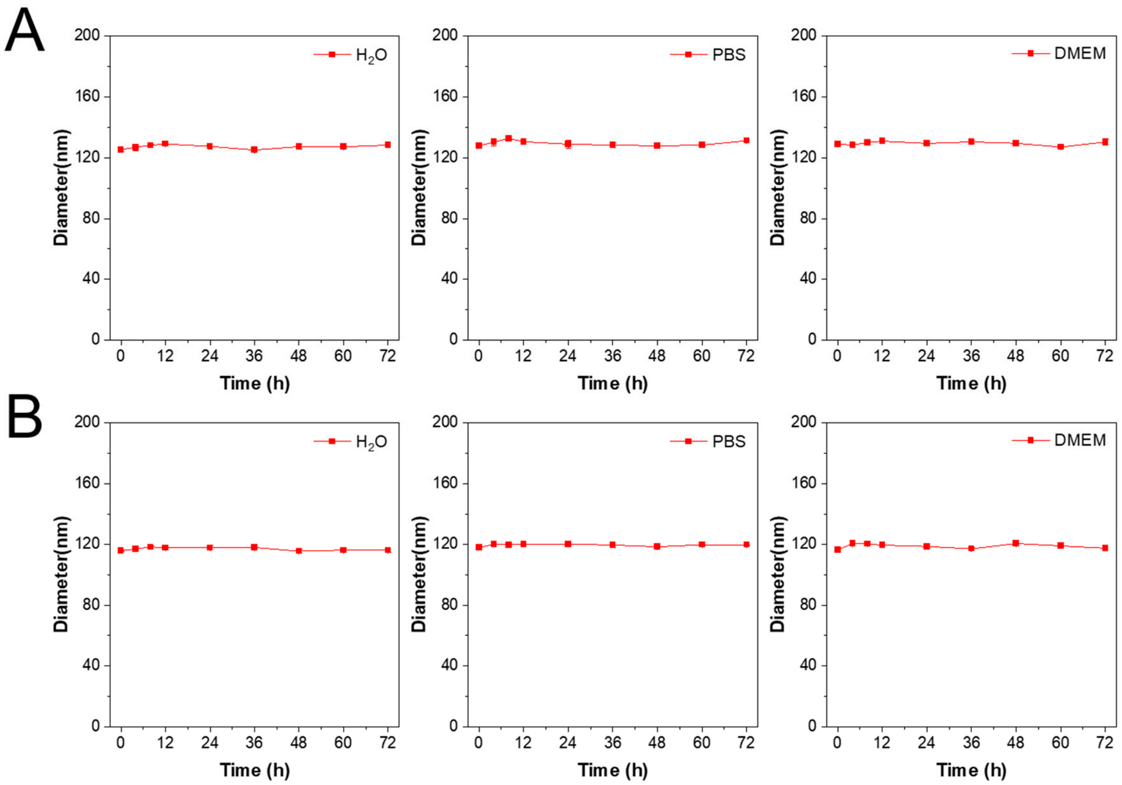

Figure S17. Stability test of (A) TCF NPs and (B) TCFBr NPs under different biological medium.
